# Bridging accessibility gaps in urban community-based basic older adult care: a comprehensive framework validated in Xi’an, China

**DOI:** 10.3389/fpubh.2025.1535987

**Published:** 2025-02-26

**Authors:** Yuyuan Zhang, Ming Zhou, Jinrong Hu, Ruoying Wang

**Affiliations:** ^1^School of Public Administration, Northwest University, Xi'an, China; ^2^School of Public Administration, Xi’an University of Architecture and Technology, Xi'an, China

**Keywords:** accessibility, basic older adult care, urban community, evaluation framework, service equity

## Abstract

**Objectives:**

Accessibility is a critical factor in ensuring equitable public services. In urban older adult care systems, resource allocation and service disparities present unique challenges. The classical “5A” theory—availability, accessibility, affordability, adaptability, and acceptability—provides a robust framework for evaluating service delivery. However, its application in urban older adult care, especially in rapidly aging societies like China, remains limited. This study aims to develop and validate a framework to address affordability, resource allocation, and service mismatches in urban older adult care systems.

**Methods:**

A web-based cross-sectional study was performed in 2023. A multi-phase methodology was adopted to construct the framework, grounded in the “5A” theory. Indicators were refined through expert consultations using the Delphi method, involving 20 experts, while the entropy weight method ensured objective indicator weighting. The framework was empirically validated in Xi’an, China, using survey data collected from 438 older adult residents across urban strata. A fuzzy comprehensive evaluation (FCE) method was employed to assess accessibility and identify key service gaps.

**Results:**

This study constructs a comprehensive evaluation framework for basic older adult care services (BECS), structured around 5 primary dimensions, 14 sary indicators, and 37 tertiary indicators. Empirical validation in Xi’an further demonstrates the framework’s scientific rigor and practical applicability. While the framework identifies strong spatial accessibility (3.8815), it also reveals critical gaps in affordability (3.1347) and psychological care (3.0862), confirming its effectiveness in diagnosing systemic disparities and guiding policy interventions.

**Conclusion:**

This study introduces a novel accessibility evaluation framework tailored for basic older adult care services, addressing critical gaps in affordability, psychological care, and service responsiveness. Empirical results validate the framework’s practicality and its alignment with the real-world conditions of urban aging societies. Furthermore, an innovative “Matching-Realization-Satisfaction” improvement pathway is proposed, offering actionable strategies to enhance accessibility and optimize service delivery. This framework serves as a replicable model for advancing equitable older adult care in rapidly aging urban communities.

## 1 Introduction

Population aging remains a persistent global challenge (1). Population aging is not limited to developed countries; developing nations are also facing this demographic trend (2). By 2030, the global population aged 65 and older is expected to reach 994 million, and this figure is projected to rise to 1.6 billion by 2050 (3). Aging has become one of the most significant demographic trends of the 21st century (4) and will shape China’s future demographic structure. According to the United Nations, these shifts highlight the growing urgency of addressing challenges posed by an aging population. By the end of 2023, individuals aged 60 and 65 and above are expected to account for 21.1 and 15.4% of China’s total population, respectively (5). The escalating aging trend poses significant challenges to the nation’s pension service system (6).

In the context of population aging and rapid social and economic transformations, expectations and preferences for living arrangements have undergone significant changes in recent decades, particularly in urban areas. The one-child policy, coupled with the rising participation of women in the workforce, has limited the younger generation’s ability to care for the older adult (7). Consequently, Chinese society has sought alternative sources of older adult care support (8), with basic older adult care services emerging as the cornerstone of the country’s multi-tiered older adult care system (9, 10). The release of Opinions on *Promoting the Construction of a BECS System* has established a strategic framework emphasizing material assistance and care services as its core components. Specifically, BECS includes essential services such as home-based care, which provides daily living support, healthcare, and in-home medical care; institutional care, which offers comprehensive long-term care and rehabilitation in dedicated facilities; health management, such as regular health check-ups, chronic disease management, and psychological health services; social participation and cultural engagement, which encourage older adult individuals to participate in community activities and foster intergenerational interactions; and policy support, such as financial subsidies and housing assistance tailored to the needs of disadvantaged groups. Despite significant progress in building its basic older adult care system, challenges persist, including urban–rural disparities, unequal resource distribution, and mismatches between service supply and demand (11), particularly in bridging the “last mile.” Despite significant progress, challenges remain in China’s basic older adult care system, such as urban–rural disparities, unequal resource distribution, and mismatches between service supply and demand, especially in addressing the “last mile.”

Accessibility is a key requirement for building a modern Chinese-style public service system (12). Enhancing accessibility has become a critical policy objective for advancing the basic public service system. Accessibility focuses on the endpoints of service delivery, aiming to improve recipients’ sense of access and satisfaction by enhancing the convenience and usability of public services, with greater emphasis on functional value. Ensuring that basic old-age services truly benefit older adult populations, while enhancing accessibility, is a pressing issue at this stage of service development. Despite progress in constructing a basic old-age care system across China, the lack of standardized evaluation metrics and frameworks persists.

We address the theoretical and practical demands of developing BECS by incorporating Western classical accessibility concepts and domestic research paradigms. First, we clarifiy the core elements and dimensions of basic older adult care service accessibility. Second, it employs the Delphi method, entropy weight method, and other approaches to construct and refine the evaluation index system for assessing the accessibility of BECS in urban communities. Representative areas are selected for empirical research to validate the system’s rationality and feasibility. Finally, the evaluation results and current practices are analyzed to pinpoint key areas and priorities for improving the accessibility of basic older adult care services.

## 2 Materials and methods

### 2.1 Study design

**Figure 1:**
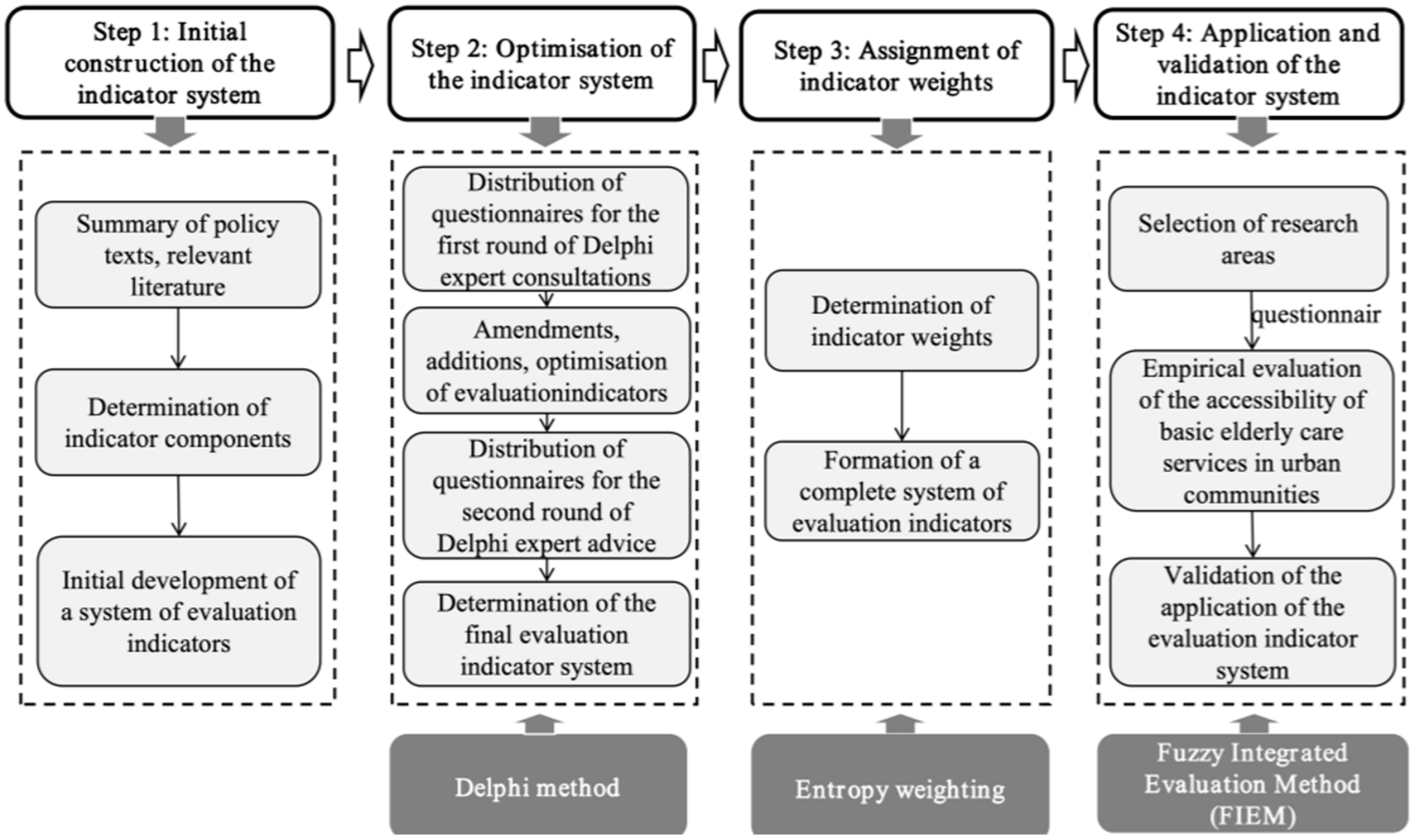
Research framework.

We employed a cross-sectional design to evaluate disparities in the accessibility of basic older adult care services (BECS) in urban districts of Xi’an, using a multidimensional framework grounded in the ‘5A’ theory: availability, accessibility, affordability, adaptability, and acceptability. This evaluation follows a systematic approach encompassing four stages: framework construction, indicator optimization, stakeholder empowerment, and application validation, as illustrated in Figure 1.

Stage 1: Initial Construction of the Indicator System—A systematic review and integration of relevant literature identifies core dimensions and detailed indicators for evaluating BECS in urban communities.
Stage 2: Optimization of the Indicator System—Indicators were developed using a structured literature review and expert consultations conducted through the Delphi method. This iterative process involved experts from gerontology, public health, and social welfare fields. A total of 5 primary dimensions, 14 sary indicators, and 37 tertiary indicators were finalized to comprehensively evaluate accessibility.
Stage 3: Indicator Weight Assignment—Weights are assigned using the entropy weighting method based on field research data, ensuring objectivity and reliability in the optimized indicator system.
Stage 4: Application and Validation of the Indicator System—A typical urban area in Xi’an is selected for empirical research. A Likert five-point scale and the fuzzy comprehensive evaluation method are employed to validate the system’s effectiveness.

### 2.2 Conceptual model of accessibility of BECS in urban communities

Accessibility refers to the actual use of services by individuals in need rather than merely the presence of facilities, indicating the extent to which public service systems are effectively utilized (13). The concept of accessibility, originating in Western healthcare (14), is discussed in academia through two primary perspectives: service utilization and the degree of “fit.” From the “service use” perspective, Andersen defines accessibility as individuals’ actual utilization of health services, emphasizing the factors that either facilitate or hinder service use (15). Conversely, Penchansky and Thomas adopt a “fit” perspective, criticizing Andersen’s exclusion of payment ability and defining accessibility as the degree of alignment between user needs and service systems (16). The matching perspective is widely recognized, forming the basis for classical models such as Katarina’s “4A” framework (17) and Penchansky and Thomas’s five-dimensional framework of accessibility, adaptability, affordability, availability, and acceptability (18). In older adult care, accessibility theory primarily evaluates service quality, often emphasizing the fit between services and user needs (19). Scholars have assessed older adult services through content, geographic and temporal, and economic accessibility. Others have refined these dimensions to construct models focusing on financial, content, and service mode accessibility (20). The “5A” model has also been widely adopted to evaluate older adult service quality comprehensively (21–23). Improving the accessibility of BECS to benefit the older adult population has emerged as a critical issue at the current stage of development.

**Figure 2:**
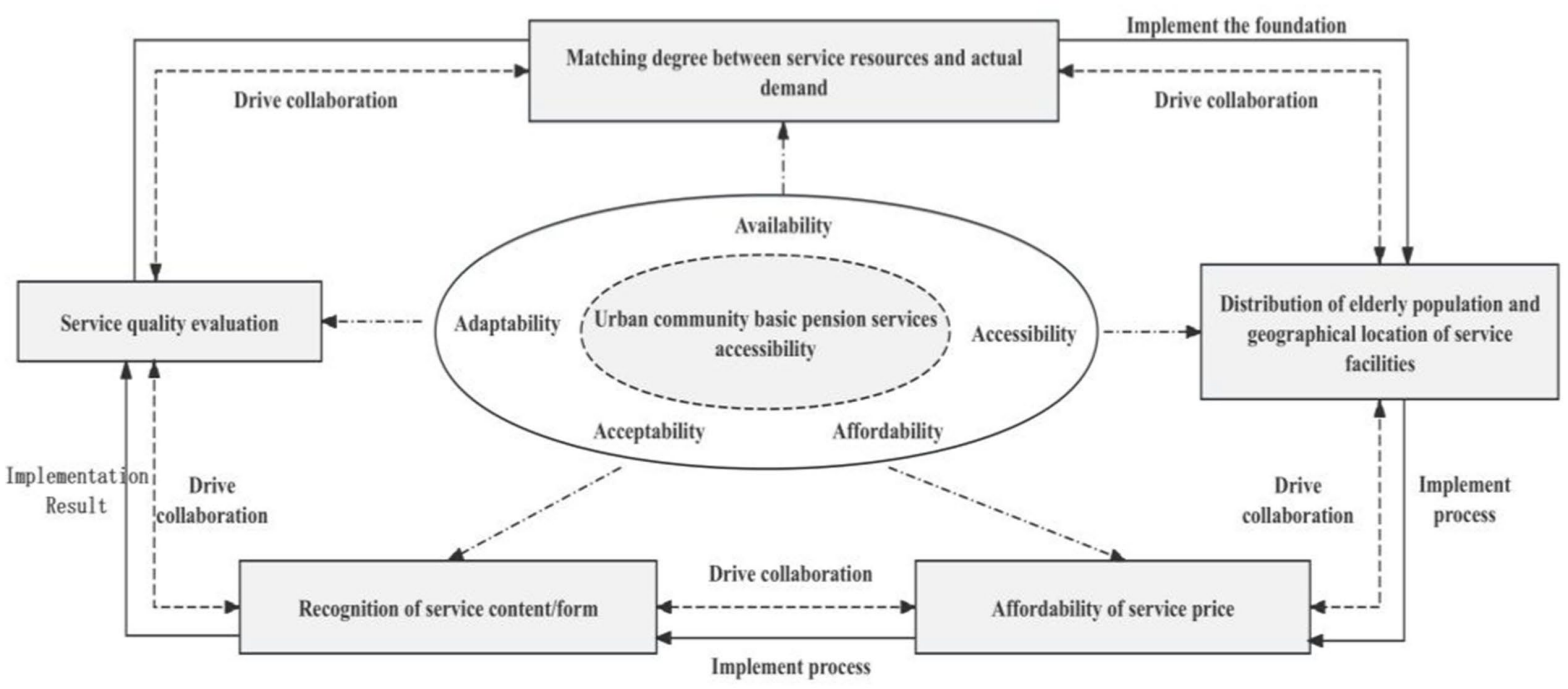
Conceptual model of ‘5A’ accessibility of basic older adult services.

Following the structural framework of “Definition-Assessment-Application,” we define the accessibility of BECS as the degree of alignment between the needs of older adult individuals and the resources of the basic older adult care system. Specifically, it examines whether older adult individuals can access government- and community-provided care services adequately, conveniently, and efficiently and whether the available resources meet service demands. Considering the development and current practices of BECS in China’s urban communities, we incorporate Penchansky and Thomas’s “5A” analysis framework of health service accessibility. The five key dimensions—availability, accessibility, affordability, adaptability, and acceptability—are adapted to the BECS system. These dimensions are further refined and extended to construct the “5A” conceptual model of accessibility for BECS in urban communities, as illustrated in Figure 2.

### 2.3 Optimization of the indicator system based on the Delphi method

#### 2.3.1 Questionnaire design

The Delphi Method is a research approach that gathers expert opinions through multiple rounds of structured correspondence to reach a consensus (24). The expert questionnaire in this study consists of two main sections: (1) This section collects basic information about the respondents, including their academic background, knowledge of basic older adult care services, and the rationale for completing the questionnaire. (2) This section asks respondents to rate the importance of indicators on a five-point Likert scale, ranging from 1 (least important) to 5 (most important). The indicator system in the questionnaire is old far based mainly on the accessibility theory and combined with national policy documents (see Supplementary material for details).

#### 2.3.2 Expert selection

Based on the study requirements, 20 experts from relevant fields were invited to participate, as detailed in Table 1. The selection criteria for these experts were: (a) holding professional titles of intermediate level or higher; (b) having a deep understanding of the field with over 10 years of relevant experience; (c) possessing an undergraduate degree or higher academic qualification; (d) demonstrating willingness and availability to engage in this study actively. The co-ordination coefficients and statistical results of the two rounds of expert consultation are detailed in Supplementary material. The co-ordination coefficients for the first and second rounds are 0.328 and 0.332 respectively, and the tests of significance satisfy the statistical requirements, demonstrating the consistency of the expert opinions.

**Table 1:** Basic information on experts.

Project title	Categorization	Numbers	Component ratio (%)
Work unit	University/Research Institutions	7	35
	Government departments	4	20
	Institutions	3	15
	Pension organizations	6	30
Area of expertise	social security	6	30
	Older adult care	7	35
	Older adult services	2	10
	Administration	5	25
Years of specialization	Less than 10 years	6	30
	10–15 years	8	40
	More than 15 years	6	30
Professional designation	Intermediate	6	30
	Deputy senior	10	50
	Full senior	4	20

### 2.4 Determination of evaluation index weights based on the entropy weight method

The accessibility of BECS in urban communities is often assessed based on the subjective perceptions of the older adult. However, traditional subjective weighting methods have inherent limitations. Therefore, we employ the entropy weight method as an objective approach to assign weights for analysis (25). The detailed process of weight calculation is as follows:

(a)   Data normalization.

Assuming that there are *n* evaluation objects and m evaluation indicators, the value of the jth indicator of the ith evaluation object is denoted as x_ij_. Through data normalization, the differences arising from different scales of the indicators are eliminated in order to make the values of the indicators have the same scale and magnitude, so as to achieve the homogeneity of the values.

For positive indicators:


(1) $$x_{ij}=\frac{X_{ij}-\min\left(X_{1j,}X_{2j,\ldots ,}X_{nj}\right)}{\max\left(X_{1j,}X_{2j,\ldots ,}X_{nj}\right)-\min\left(X_{1j,}X_{2j,\ldots ,}X_{nj}\right)}$$

For negative indicators:


(2) $$x_{ij}=\frac{\max\left(X_{1j,}X_{2j,\ldots ,}X_{nj}\right)-X_{ij}}{\max\left(X_{1j,}X_{2j,\ldots ,}X_{nj}\right)-\min\left(X_{1j,}X_{2j,\ldots ,}X_{nj}\right)}$$

(b)   Calculation of the weight of the ith evaluation object under the jth evaluation indicator:

(3) $$P_{ij}=\frac{X_{\mathrm{ij}}}{\sum_{i=1}^{n}X_{\mathrm{ij}}}$$

(c)   Calculate the entropy value of the jth indicator:

(4) $$e_{j}=-k\overset{n}{\underset{i=1}{\sum }}p_{ij}\ln\left(p_{ij}\right),j=1,\ldots ,m$$

(d)   Calculate the information entropy redundancy (utility value):

(5) $$d_{j}=1-e_{j},j=1,\ldots ,m$$

(e)   Calculation of the weights of the indicators:

(6) $$w_{j}=\frac{d_{j}}{\sum_{j=1}^{m}d_{j}},j=1,\ldots ,m$$

Following the outlined process, the weights of the second-level indicators are calculated as the sum of the corresponding third-level indicator weights. Similarly, the weights of the first-level indicators are derived by aggregating the weights of the second-level indicators. The calculation of these weights is based on Equations 1–7, which detail the normalization and weighting procedures. The specific weight results, which will be presented later in Table 5, outline the accessibility evaluation indicator system and its associated weights for basic older adult services in urban communities. These results are detailed in the Results section (Section 3.2).

### 2.5 Application of the evaluation index system based on the fuzzy comprehensive evaluation method

#### 2.5.1 Evaluation process

The fuzzy comprehensive evaluation method applies fuzzy mathematical theory to address evaluation factors with ambiguous boundaries and challenges in quantification (26). The fuzzy comprehensive evaluation (FCE) method was employed to integrate both qualitative and quantitative data for each dimension. This approach accommodates uncertainty and subjectivity inherent in survey responses, synthesizing scores to generate an overall accessibility evaluation. In addition to the structured Likert-scale questions providing quantitative inputs, qualitative data were also incorporated into the FCE analysis. Examples of qualitative data include: (1) Open-ended survey questions: Participants provided detailed feedback on their satisfaction with cultural activities, emotional support services, and accessibility of community facilities. These responses supplemented quantitative ratings and offered insights into unmet needs. (2) Field observations: During visits to community service centers, researchers documented observations related to facility usability, inclusiveness of service environments, and staff responsiveness, which informed the evaluation of adaptability and acceptability dimensions. (3) Caregiver narratives: For participants unable to complete the survey independently, caregivers contributed qualitative insights regarding the challenges and effectiveness of home-based care services.

The FCE process was employed to assess the accessibility of Xi’an’s central city through the following steps:

1. Construct the evaluation factor set. Denote the set of indicators for this evaluation of the accessibility of BECS in urban communities by U, U = {u1, u2, …, um}.
2. Construct the evaluation set. The evaluation set is the result synthesized by the respondents’ scores on the evaluation of the accessibility of basic older adult care services. The evaluation ratings are described by V = {V1, V2, …, V5}, where V1 means very satisfied, V2 means satisfied, V3 means average, V4 means dissatisfied, and V5 means very dissatisfied.
3. Determining the degree of affiliation. The number of samples corresponding to the evaluation levels of the 37 tertiary indicators can be obtained according to the scoring situation, divided by the total number of the corresponding, that is, to obtain the degree of affiliation corresponding to each level.
4. Construct the weight set. The weight of each indicator has been calculated according to the entropy weight method.
5. Compound operation of fuzzy matrix, through the synthetic operation of weight set W and evaluation result V, in order to precise the overall judgment vector of each indicator, and get the evaluation result corresponding to each indicator.

#### 2.5.2 Selection of evaluation objects

Xi’an, the capital of Shaanxi Province, was chosen as the study site due to its unique demographic profile and its significance in addressing aging-related challenges in urban China. As of 2023, Xi’an has a population of approximately 13.08 million, with 15.5% aged 60 or older and 12.6% aged 65 or older (27), classifying it as a deeply aging society. The city has faced aging-related issues, such as an imbalance between the supply and demand for older adult care services, earlier than many other Chinese cities. Its advanced efforts in developing older adult care infrastructure and services make Xi’an an ideal location to test the feasibility and applicability of the proposed evaluation framework.

#### 2.5.3 Questionnaire distribution and data collection

We focused on the central urban areas of Xi’an, including Xincheng, Beilin, Lianhu, Baqiao, Weiyang, and Yanta districts, as the sample region. A combination of stratified and random sampling was employed to ensure a diverse and representative sample across different urban strata. Eligible participants were required to meet the following criteria: (1) aged 60 years or older and (2) had previously received basic older adult care services.

Data collection was primarily conducted at community older adult service centers through face-to-face interviews and paper questionnaires, complemented by surveys in other community locations, such as activity centers and nearby parks. For participants with limited mobility or reduced self-care ability, caregivers or family members were permitted to complete the questionnaire on their behalf. This stratified sampling approach, combined with the targeted distribution of questionnaires, ensured the validity and authenticity of the responses while reflecting the diverse conditions of older adult residents in urban areas.

To determine the sample size, we applied the formula for large sample populations proposed by Wu (28):


(7) $$n\geq {\left(\frac{k}{\alpha }\right)}^{2}p\left(1-P\right)$$

where the significant level *α* is 0.05 (28) and the confidence level used for interval estimation is 1-*α* = 0.95, at which point the quantile *k* = 1.96, and according to this formula, the calculation yields *n* ≥ 384.

To account for potential non-responses and ensure a robust sample size, a total of 438 questionnaires were distributed. Among these, 430 valid responses were recovered, resulting in a response rate of 98.17%. Missing data accounted for less than 5% of the total dataset, primarily due to incomplete demographic information. These missing values were addressed using multiple imputation to ensure data integrity without biasing the analysis. The respondents’ basic information is detailed in Table 2.

**Table 2:** Basic information of survey respondents.

Basic Information	Form	Percentage (%)
Genders	Male	51.85%
	Female	48.15%
Age	60–64 years	23.84%
	65–69 years	27.08%
	70–74 years old	24.31%
	75 and above	24.77%
Income situation	0–999 Yuan	16.67%
	1000–1999 Yuan	29.63%
	2000–3999 Yuan	41.90%
	4000Yuan and above	11.81%
Health situation	Health	23.84%
	Good	40.05%
	Fair	27.78%
	Unhealthy	8.33%
Residential situation	Living with spouse	42.13%
	Living with children	21.53%
	Living alone	19.68%
	Living with spouse and children	14.35%
	Other	2.31%

## 3 Results

### 3.1 Analysis of questionnaire data for the Delphi method

#### 3.1.1 Degree of expert authority

Expert authority degree coefficient (Cr) ≥ 0.70 is acceptable coefficient (29), expert authority coefficient (Cr) = (Ca + Cs) / 2, Ca is the basis of expert judgment. The Cr value ranges from 0 to 1, with higher values indicating greater expert authority. The quantitative results for expert authority are presented in Table 3.

**Table 3:** Degree of authority of experts.

Rounds	Coefficient of appreciation (Ca)	Degree of familiarity(Cs)	Authority factor (Cr)
Round 1	0.91	0.85	0.88
Round 2	0.96	0.90	0.93

#### 3.1.2 Degree of coherence of expert opinions

The consistency of the evaluation indicators is assessed using Kendall’s WWW harmony coefficient, which ranges from 0 to 1. Higher WWW values indicate greater consistency among expert opinions. As shown in Table 4, the significance test for the harmony coefficient yielded a *p*-value below the threshold of 0.05, confirming its statistical significance. Therefore, the expert evaluations demonstrated consistency and satisfied the criteria for indicator screening.

**Table 4:** Degree of coordination between the two rounds of consultancies.

Degree of expert coordination		Entry	Kendall’s W	*χ* ^2^	*p*
First round of consultations	Overall	58	0.328	373.792	0.000
	First level indicator	5	0.217	17.333	0.002
	Secondary indicators	14	0.113	29.263	0.006
	Third level indicators	39	0.390	296.663	0.000
Second round of consultations	Overall	56	0.332	365.012	0.000
	First level indicator	5	0.125	10.000	0.040
	Secondary indicators	14	0.246	64.059	0.000
	Third level indicators	37	0.307	220.906	0.000

### 3.2 Construction of evaluation indicator system for accessibility of basic older adult services in urban communities

Following two rounds of Delphi expert consultation, a final evaluation indicator system for assessing the accessibility of BECS in urban communities was established. The system comprises 5 first-level indicators, 14 second-level indicators, and 37 third-level indicators, as detailed in Table 5. According to the weighting order presented in Table 5, affordability ranks highest, followed by availability, adaptability, acceptability, and accessibility. Among the 14 second-level indicators, resource provision emerges as the core element with the highest weight (0.0943), underscoring the critical role of abundant resources in ensuring access to older adult care services. Facility provision (0.077) and facility layout (0.0616) rank next, emphasizing the significance of sufficient older adult beds and diverse programs in meeting the varied needs of the older adult population. The prices of life care services (0.0820) and spiritual comfort services (0.0859) carry comparable weights, reflecting the older adult’s dual focus on quality of life and emotional well-being.

**Table 5:** Evaluation indicator system for accessibility of basic older adult services in urban communities and its weights.

First level indicators	Weight	Secondary indicators	Weight	Third level indicators	Weight
A Availability	0.2329	A1 Facility layout	0.0616	A11 Number of community older adult service centers	0.0311
				A12 Building area of community older adult care service center	0.0305
		A2 Facility configuration	0.0770	A21 Number of beds in community older adult service centers	0.0464
				A22 Number of service facilities in community older adult care service centers	0.0306
		A3 Resource supply	0.0943	A31 Types of older adult services provided by community older adult service centers	0.0333
				A32 The number of older adult care service projects provided by community older adult care service centers	0.0304
				A33 Number of service personnel in community older adult service centers	0.0306
B Accessibility	0.0637	B1Space reachable	0.0283	B11 Distance from residence to community older adult care service center	0.0127
				B12 The convenience level from the residence to the community older adult care service center	0.0156
		B2Time achievable	0.0354	B21 Waiting time for nursing staff’s on-site service	0.0184
				B22Time consumption from residence to community older adult care service center	0.0170
C Affordability	0.3161	C1 Affordability of prices for obtaining life care services	0.0820	C11 Meal assistance service	0.0202
				C12 Cleaning assistance service	0.0243
				C13 Agency service	0.0375
		C2 Affordable access to medical care services	0.1006	C21 Rehabilitation nursing services	0.0334
				C22 Health management services	0.0293
				C23 Healthcare Services	0.0379
		C3 Affordability of prices for obtaining mental comfort services	0.0859	C31 Emotional Communication Services	0.0444
				C32 Psychological counseling services	0.0415
		C4 Affordability of prices for cultural and entertainment services	0.0476	C41 Entertainment	0.0178
				C42 Education for the older adult	0.0298
D Acceptability	0.1366	D1 Acceptance of service content	0.0907	D11 Acceptance of life-care services	0.0224
				D12 Acceptance of medical care services	0.0193
				D13 Acceptance of mental comfort services	0.0267
				D14 Acceptance of cultural and recreational services	0.0223
		D2 Acceptance of service modalities	0.0459	D21 Acceptance of the home-based care (in-home) service approach	0.0274
				D22 Acceptance of daycare (day care) services	0.0185
E Adaptability	0.2507	E1 Satisfaction with facility construction	0.0736	E11Satisfaction with the layout of community older adult service facilities	0.0245
				E12 Satisfaction with the provision of community older adult service facilities	0.0245
				E13 Satisfaction with the aging-friendly construction of community older adult service centers (stations)	0.0246
		E2Satisfaction with quality of service	0.1318	E21 Satisfaction with life care services provided in the community	0.0244
				E22 Satisfaction with health care services provided in the community	0.0250
				E23 Satisfaction with mental comfort services provided in the community	0.0352
				E24 Satisfaction with cultural and recreational services provided by the community	0.0204
				E25 Satisfaction with service personnel	0.0268
		E3 Satisfaction with the service environment	0.0453	E31 Satisfaction with the internal environment of community older adult service centers	0.0244
				E32 Satisfaction with age-friendly environment in the community	0.0209

### 3.3 Application of the evaluation index system for accessibility of BECS in urban communities

The primary, secondary, and tertiary indicators, along with the comprehensive evaluation results, were determined by de-fuzzifying the evaluation result set, as detailed in Table 6. Using the final calculated PPP value for de-fuzzification as an example, the comprehensive evaluation score for the accessibility of basic community older adult care services in Xi’an’s urban center is:

**Table 6:** Evaluation results of the accessibility of basic community-based older adult care services in the six urban districts of Xi’an City.

First level indicators	Score	Secondary indicators	Score	Third level indicators	Score
A	3.1544	A1	3.1542	A11	3.1442
				A12	3.1651
		A2	3.0816	A21	2.9790
				A22	3.2372
		A3	3.2135	A31	3.2071
				A32	3.2234
				A33	3.2114
B	3.6724	B1	3.8429	B11	3.8815
				B12	3.8116
		B2	3.5369	B21	3.4767
				B22	3.6022
C	3.2668	C1	3.4173	C11	3.6675
				C12	3.3744
				C13	3.3099
		C2	3.2142	C21	3.2303
				C22	3.2976
				C23	3.1347
		C3	3.0791	C31	3.0837
				C32	3.0742
		C4	3.4582	C41	3.7348
				C42	3.2930
D	3.5627	D1	3.5429	D11	3.6536
				D12	3.7349
				D13	3.2931
				D14	3.5650
		D2	3.6026	D21	3.5697
				D22	3.6511
E	3.3721	E1	3.3868	E11	3.3838
				E12	3.3652
				E13	3.4115
		E2	3.3210	E21	3.3045
				E22	3.3257
				E23	3.0862
				E24	3.4722
				E25	3.5255
		E3	3.4971	E31	3.5347
				E32	3.4535


5×0.1241+4×0.2989+3×0.4005+2×0.1390+1×0.0374=3.3330

**Figure 3:**
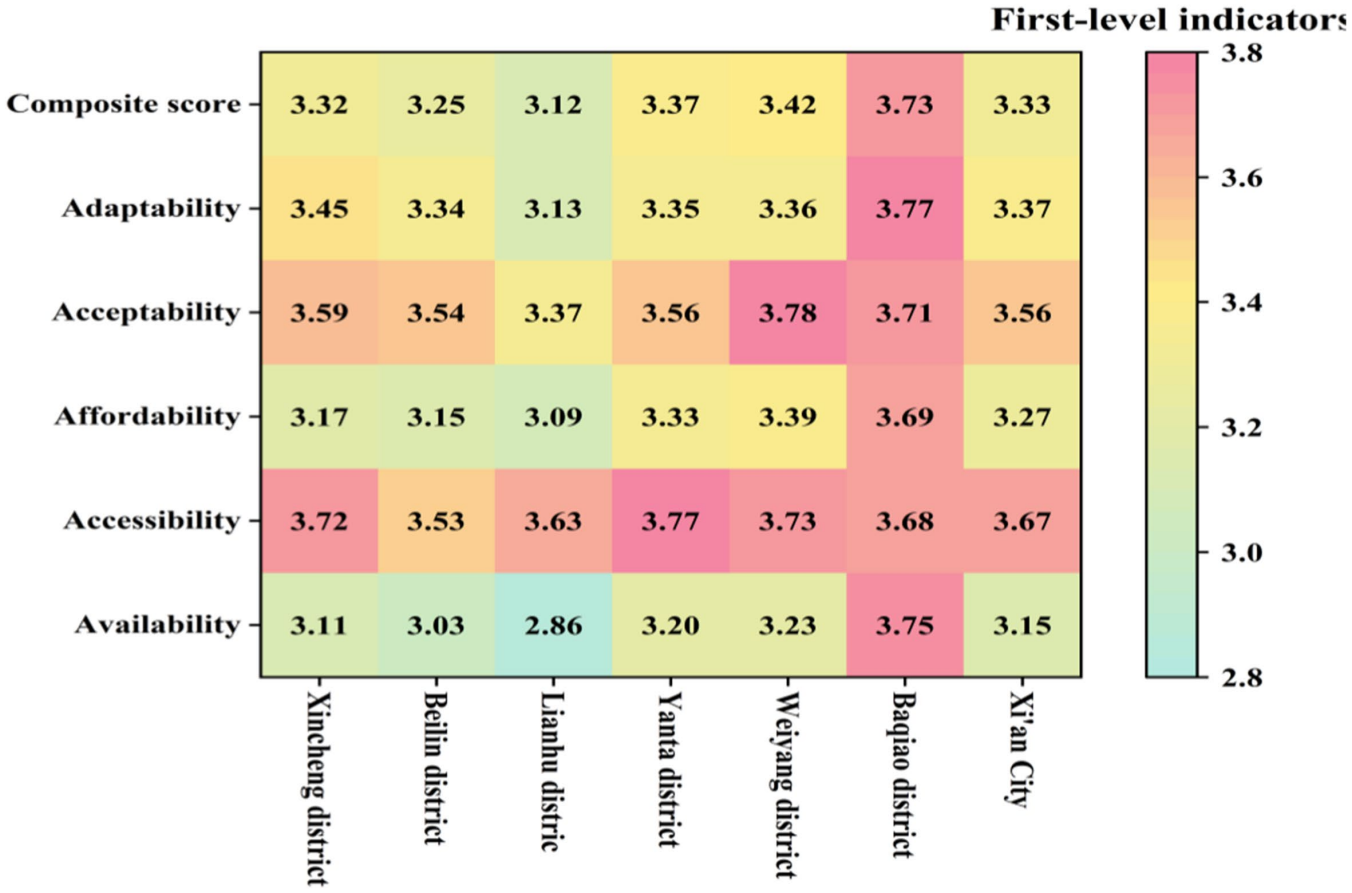
Hot map of accessibility evaluation results for BECS in urban communities.

The empirical analysis in Xian’s central urban area underscores the importance of affordability, availability, and adaptability in ensuring equitable older adult care. These findings are critical for policymakers and healthcare providers, emphasizing key areas for prioritizing resource allocation and service improvements. A systematic assessment of the accessibility of community-based older adult care services in Xi’an’s central urban districts (Baqiao, Weiyang, Yanta, Xincheng, Beilin, and Lianhu) was conducted using the fuzzy comprehensive evaluation method, with results presented in Figure 3. We result indicate that the overall accessibility score of Xi’an’s six central urban districts is 3.3330, falling within the “average” range to “good.” This reflects an overall efficient provision of public service facilities, adequately meeting the basic daily needs of most older adult residents.

The data reveal a structural imbalance between resource supply and growing demand, highlighting an availability dilemma (30). The availability score is 3.1544, with facility layout and equipment scoring 3.1542 and 3.0816, respectively, suggesting that the infrastructure generally meets the basic needs of the older adult. However, the score for the number of beds is relatively low (3.2135), revealing a structural shortfall in resource capacity. This result reflects a lag in facility investment and planning relative to the surge in service demand caused by accelerated aging.

Accessibility is challenged by both spatial optimization and service responsiveness (31). The overall accessibility score is 3.6724, with spatial accessibility scoring a relatively high 3.8429, suggesting that the central city’s service facility layout is reasonable. However, the time accessibility score is relatively low (3.5369), with waiting times for door-to-door services scoring only 3.4767, indicating significant room for improvement in service responsiveness. This disparity between spatial and temporal accessibility highlights the tension between facility centralization and service personalization. While centralized facility layouts provide spatial convenience, they limit the flexibility of service responsiveness, particularly in addressing personalized and urgent needs.

Affordability faces a dual imbalance in economic costs (32). The affordability score is 3.2668, with meal assistance services scoring the highest (3.6675) and healthcare services the lowest (3.1347). The high cost of healthcare services imposes a financial burden on low-income older adult groups, exposing a gap between basic living and healthcare needs. This finding underscores insufficient policy support and the limitations of the social security system in addressing high-cost healthcare services.

Acceptability faces challenges related to neglecting psychological needs and insufficient cultural adaptation. The acceptability score is 3.5627, with medical services scoring the highest (3.7349) and psychological comfort services the lowest (3.2931). The low score reflects insufficient psychological support services and unmet emotional and spiritual care needs among the older adult. This issue indicates an overemphasis on material needs in the older adult service system, with insufficient attention and resources allocated to soft needs such as emotional care and social participation.

Adaptability faces challenges due to the absence of a dynamic adjustment mechanism. The adaptability score is 3.3721, with high satisfaction in cultural and recreational services (3.4722) and the lowest satisfaction in spiritual comfort services (3.0862). The low satisfaction score highlights inadequate psychological support services and the absence of a dynamic adjustment mechanism to address the increasingly complex needs of the older adult. The mismatch between service supply and demand has resulted in a disconnect between service quality and the actual needs of the older adult.

These results underscore the systemic challenges in resource allocation and service capacity, necessitating policy interventions tailored to affordability and psychological care deficiencies.

## 4 Discussion

### 4.1 Comprehensive evaluation framework and key findings

We developed a comprehensive indicator system to assess the accessibility of basic older adult care services (BECS) in urban communities, structured around five dimensions: availability, accessibility, affordability, adaptability, and acceptability. Using Xi’an City as a case study, the framework highlights critical disparities in service provision. For instance, affordability received a score of 3.1347, indicating significant financial barriers for older adult residents, while spiritual comfort services scored 3.0862, revealing substantial gaps in emotional and psychological care. Conversely, spatial accessibility achieved a high score of 3.8815, reflecting effective infrastructure planning and facility distribution. However, delays in caregiver home visits, with a score of 3.4767, indicate persistent gaps in responsiveness and personalized care. These results underscore the multidimensional challenges facing urban older adult care systems and provide actionable insights for addressing them.

### 4.2 Unique characteristics of BECS and policy implications

A distinguishing feature of this study is its focus on the unique characteristics of BECS, emphasizing universal coverage and equity. BECS prioritize economically disadvantaged and health-compromised populations, contrasting with general older adult care frameworks that often target affluent groups with premium or personalized services. The results validate this distinction, as affordability and availability emerge as key dimensions requiring targeted interventions. The low affordability score of 3.1347 highlights the urgent need for financial subsidies, particularly for low-income older adult populations. The framework’s emphasis on equity and inclusivity makes it a practical tool for guiding public welfare policy.

### 4.3 Systemic challenges and strategic solutions

As a deeply aging city, Xi’an faces systemic constraints such as resource imbalances and insufficient service capacity. While investments in infrastructure have enhanced spatial accessibility, affordability and psychological care deficiencies remain significant barriers. For example, while investments in infrastructure have enhanced spatial accessibility (score: 3.8815), persistent affordability challenges and gaps in psychological care (score: 3.0862) underscore the need for targeted investments. Addressing these challenges requires a multi-pronged strategy.

**Figure 4:**
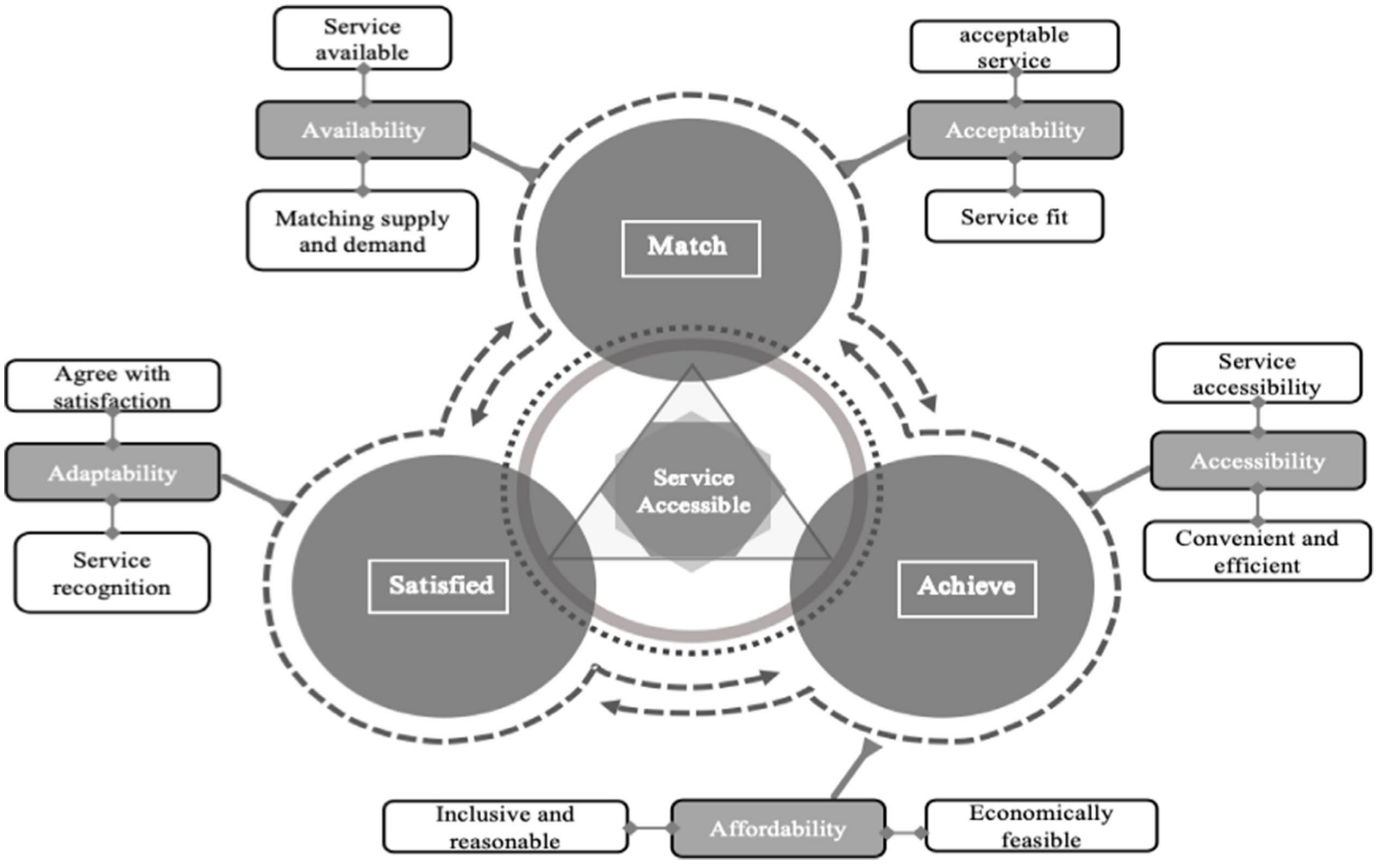
Pathway for improving the accessibility of BECS in urban communities.

Based on these findings, We adopt a “demand–supply matching, service realization, and satisfaction enhancement” framework to examine strategies for improving the accessibility of BECS in urban communities (Figure 4). Effective demand–supply alignment requires a dynamic, data-driven approach to monitor demographic trends and forecast resource needs. Transitioning from reactive to proactive service models involves leveraging intelligent platforms to enable real-time resource allocation and optimize service delivery. Service realization depends on integrated funding mechanisms and age-friendly environments. A unified funding framework ensures efficient resource utilization, while investments in accessible infrastructure, including barrier-free facilities and digital adaptations, address the diverse needs of older adult populations. Enhancing satisfaction is critical for fostering continuous improvement, supported by comprehensive oversight mechanisms and innovative service delivery approaches. Comprehensive monitoring frameworks—incorporating governmental, non-governmental, and public stakeholders—facilitate accountability, while feedback systems inform actionable improvements. Innovative models, such as ‘Internet + Older adult Care’ and mutual assistance initiatives like ‘time banking,’ expand service options and enhance the well-being of older adults. Collectively, these strategies offer a holistic and adaptable blueprint for advancing the accessibility and equity of urban older adult care services.

### 4.4 Framework validation and policy implications

The validation in Xi’an confirms the framework’s effectiveness in assessing and enhancing the accessibility of basic older adult care services. While spatial accessibility is strong (3.8815), affordability and psychological care remain key challenges, requiring targeted policy actions. To improve affordability, expanding financial support, such as subsidies for low-income older adult individuals and creating a long-term care security system, is essential. To address service adaptability, digital platforms like “Internet + Older adult Care” should be developed to integrate home-based care, telemedicine, and community mental health services, improving resource allocation and service efficiency. Strengthening home and community care, through home adaptations and family doctor services, will enhance the synergy between family and professional care.

In the long term, building a robust basic older adult care system must support equitable public services. By reducing accessibility and affordability gaps, governments can facilitate the transition from basic care to higher-quality, personalized services, promoting fairness and improving the well-being of older adult individuals in urban environments.

### 4.5 Limitations and future research directions

This study has certain limitations. First, the empirical analysis is confined to Xi’an City, which may restrict the generalizability of the findings. Future research should expand to additional cities or regions and conduct comparative analyses to validate the framework’s broader applicability. Second, the study primarily uses quantitative methods, lacking an in-depth exploration of the older adult’s subjective experiences and emotional needs. Future research could address this gap by incorporating qualitative methods to capture the psychological experiences and satisfaction evaluations of older adult individuals. Lastly, with the accelerating integration of artificial intelligence and digital platforms into older adult care, future research should explore their applications in service delivery and accessibility assessments, providing insights for developing older adult care systems in the new era.

## 5 Conclusion

This study introduces a novel framework for evaluating the accessibility of basic older adult care services (BECS), addressing critical dimensions such as availability, affordability, adaptability, and acceptability. Grounded in the “5A” theory, the framework was empirically validated in Xi’an, China, and revealed significant disparities in affordability and psychological care services, alongside strong performance in spatial accessibility. These findings underscore the urgent need for targeted policy interventions to address resource imbalances.

The proposed “Matching-Realization-Satisfaction” pathway provides targeted strategies, including demand–supply alignment and innovative service models, to enhance BECS accessibility. By leveraging dynamic demand–supply alignment, integrated funding mechanisms, and innovative service delivery models.

Despite its contributions, this study is limited by its focus on Xi’an City and its primary use of quantitative methods, which may not fully capture subjective experiences. Future research should expand the framework’s application to diverse urban contexts and incorporate qualitative approaches to capture the subjective experiences of older adult individuals. Additionally, exploring the integration of artificial intelligence and digital platforms in service delivery could further enhance the framework’s adaptability in the digital age.

This study advances the theoretical and practical understanding of older adult care accessibility, providing a scalable and adaptable framework to inform policy development and service optimization. By addressing the complex challenges of affordability, psychological care, and responsiveness, it contributes to building equitable and sustainable older adult care systems globally.

## Data Availability

The original contributions presented in the study are included in the article/Supplementary material, further inquiries can be directed to the corresponding author.
