# Supplementary material for: Bridging accessibility gaps in urban community-based basic older adult care: a comprehensive framework validated in Xi’an, China
Source: Front Public Health. 2025 Feb 26;13:1535987. doi: 10.3389/fpubh.2025.1535987 (PMC11896861; doi:10.3389/fpubh.2025.1535987)
Supplement: Supplementary file 1 [file Table_1.docx]

Supplementary Material

# 1 Basis of indicator selection

The evaluation index system for basic elderly services in urban communities is based on the deconstruction of relevant policies through the lens of accessibility theory, establishing a structure of evaluation elements grounded in theoretical principles.Accessibility is subdivided into five primary dimensions: availability, affordability, adaptability, acceptability, and spatial accessibility. "The system’s academic foundation is established through a comprehensive review of literature on service accessibility and elderly care, as well as key policy documents such as the ‘14th Five-Year Plan for the Development of the National Elderly and the Elderly Service System’ and the ‘Opinions on Promoting the Construction of the Basic Elderly Service System,’ ensuring alignment with national policy implementation goals, the specific sources of the evaluation indicators for the accessibility of the basic elderly services in the urban community are shown in Table 1.

***Table 1*** Sources and Dimensions of Evaluation Indicators for Basic Elderly Care Accessibility

| **Evaluation Dimension** | **Evaluation Dimension** | **Selection criteria for indicators** |
| --- | --- | --- |
| A Availability | A1 Facility Layout | Guidance on Strengthening the Planning and Construction of Elderly Service Facilities，Haoli et al（2015）[^[[1]](#footnote-0)^] |
|  | A2 Facility configuration | National Plan for the Development of the Elderly and the Elderly Service System in the Fourteenth Five-Year Plan，Yong Lan et al（2018）[^[[2]](#footnote-1)^] |
|  | A3 Resource supply | Opinions on Promoting the Construction of a Basic Elderly Service System，asic Norms for Home Care Services for the Elderly at Home（GB/T 43153-2023），Han xiaowei et al（2023）[^[[3]](#footnote-2)^]，Haoli et al（2025）[^[[4]](#footnote-3)^]，Zeng Quanhai et al（2024）[^[[5]](#footnote-4)^] |
| B Accessibility | B1 Space reachable | Opinions on Promoting the Construction of a Basic Elderly Service System，Xu Xin et al（2017）[^[[6]](#footnote-5)^],Wu Bo et al（2023）[^[[7]](#footnote-6)^] |
|  | B2 Time achievable | Gamlu et al（2023）[^[[8]](#footnote-7)^]，Li Yunhe et al（2024）[^[[9]](#footnote-8)^],Wang Zhenzhen et al（2020）[^[[10]](#footnote-9)^] |
| C Affordability | C1 Affordability of prices for obtaining life care services | Opinions on Promoting the Construction of a Basic Elderly Service System，Code of Practice for Community Aged Care Services in the Home，Wang Lijian et al（2017）[^[[11]](#footnote-10)^] |
|  | C2 Affordable access to medical care services |  |
|  | C3 Affordability of prices for obtaining mental  comfort services |  |
|  | C4 Affordability of prices for cultural and  entertainment services |  |
| D Acceptability | D1 Acceptance of service content | Maduoduo et al（2023）[^[[12]](#footnote-11)^],Zeng Quanhai et al（2024）[^[[13]](#footnote-12)^] |
|  | D2 Acceptance of service modalities |  |
| E Adaptability | E1 Satisfaction with facility construction | Opinions of the General Office of the State Council on Promoting the Development of Elderly Services,National Plan for the Development of the Elderly and the Elderly Service System in the Fourteenth Five-Year Plan，Li Yunhua et al（2024）[^[[14]](#footnote-13)^],Maduoduo et al（2023）[^[[15]](#footnote-14)^] |
|  | E2 Satisfaction with quality of service |  |
|  | E3 Satisfaction with the service environment |  |

# 2 The concentration and dispersion of the results corresponding to the first round of Delphi expert consultation

After the preliminary construction, this paper forms an evaluation index system for the accessibility of basic elderly care services in urban communities that contains a system covering 5 first-level indicators, 14 second-level indicators and 39 third-level indicators. The importance of each indicator was quantitatively assessed by adopting the Likert 5-point scale, and the specific calculation results of the importance assignment and coefficient of variation of each indicator are shown in Table 2.

***Table 2*** Concentration and dispersion of indicators in the first round of expert opinions

| **Indicator Name** | **Importance assignment（**$\bar{\mathbf{X}}$**±S）** | **coefficient of variation**  **（CV）** |
| --- | --- | --- |
| A Availability | 4.900±0.308 | 0.063 |
| A1 Facility layout | 4.900±0.308 | 0.063 |
| A11 Number of community elderly care  service centers (stations) | 4.500±0.513 | 0.114 |
| A12 Building area of community elderly care  service center (station) | 4.400±0.503 | 0.114 |
| A2 Facility configuration | 4.800±0.410 | 0.085 |
| A21 The number of beds in community elderly care service centers (stations) | 4.350±0.587 | 0.135 |
| A22 Number of service facilities in community elderly care service centers (stations) | 4.500±0.607 | 0.135 |
| **A23 Facility coverage rate of community elderly care service centers (stations)** | **1.950±0.605** | **0.310** |
| A3 Resource supply | 4.900±0.308 | 0.063 |
| A31 Types of elderly care services provided by community elderly care service centers (stations) | 4.300±0.733 | 0.170 |
| A32 The number of elderly care service projects provided by community elderly care service centers (stations) | 4.200±0.410 | 0.098 |
| **A33The ratio of nursing staff for elderly care services to the number of elderly people receiving services** | **2.200±0.768** | **0.349** |
| B Accessibility | 4.500±0.513 | 0.114 |
| B1 Space reachable | 4.650±0.489 | 0.105 |
| B11 Distance from residence to community elderly care service center (station) | 4.550±0.605 | 0.132 |
| B12 The convenience level from the residence to the community elderly care service center (station) | 4.600±0.503 | 0.109 |
| **B13 The cost of moving from the residence to the community elderly care service point** | **2.100±0.641** | **0.305** |
| B2 Time achievable | 4.600±0.503 | 0.109 |
| B21 Waiting time for nursing staff's on-site service | 4.450±0.510 | 0.115 |
| B22Time consumption from residence to community elderly care service center (station) | 4.300±0.571 | 0.133 |
| C Affordability | 4.700±0.470 | 0.100 |
| C1 Affordability of prices for obtaining life care services | 4.650±0.489 | 0.105 |
| C11 Meal assistance service | 4.450±0.510 | 0.115 |
| C12 Cleaning assistance service | 4.500±0.688 | 0.153 |
| C13 Agency service | 4.600±0.598 | 0.130 |
| C2 Affordable access to medical care services | 4.450±0.510 | 0.115 |
| C21 Rehabilitation nursing services | 4.550±0.510 | 0.112 |
| C22 Health management services | 4.600±0.503 | 0.109 |
| C23 Healthcare Services | 4.650±0.489 | 0.105 |
| C3 Affordability of prices for obtaining mental  comfort services | 4.550±0.510 | 0.112 |
| C31 Emotional Communication Services | 4.600±0.503 | 0.109 |
| **C32 Emotional counseling service** | **2.000±0.562** | **0.281** |
| C33 Psychological counseling services | 4.400±0.503 | 0.114 |
| C4 Affordability of prices for cultural and entertainment services | 4.600±0.503 | 0.109 |
| C41 Entertainment | 4.500±0.513 | 0.114 |
| C42 Education for the elderly | 4.650±0.489 | 0.105 |
| D Acceptability | 4.450±0.510 | 0.115 |
| D1 Acceptance of service content | 4.700±0.470 | 0.100 |
| D11 Acceptance of life-care services | 4.450±0.510 | 0.115 |
| D12 Acceptance of medical care services | 4.250±0.716 | 0.168 |
| D13 Acceptance of mental comfort services | 4.300±0.733 | 0.170 |
| D14 Acceptance of cultural and recreational services | 4.350±0.745 | 0.171 |
| D2 Acceptance of service modalities | 4.500±0.513 | 0.114 |
| D21 Acceptance of the home-based care (in-home)  service approach | 4.450±0.605 | 0.136 |
| D22 Acceptance of daycare (day care) services | 4.300±0.571 | 0.133 |
| E Adaptable | 4.700±0.470 | 0.100 |
| E1 Satisfaction with facility construction | 4.650±0.489 | 0.105 |
| E11Satisfaction with the layout of community  elderly service facilities | 4.400±0.503 | 0.114 |
| E12 Satisfaction with the provision of community elderly service facilities | 4.500±0.513 | 0.114 |
| E2 Satisfaction with quality of service | 4.800±0.410 | 0.085 |
| E21 Satisfaction with life care services provided  in the community | 4.700±0.470 | 0.100 |
| E22 Satisfaction with health care services provided  in the community | 4.450±0.510 | 0.115 |
| E23 Satisfaction with mental comfort services provided  in the community | 4.250±0.716 | 0.168 |
| E24 Satisfaction with cultural and recreational services provided by the community | 4.350±0.745 | 0.171 |
| E25 Satisfaction with service personnel | 4.350±0.489 | 0.112 |
| E3 Satisfaction with the service environment | 4.400±0.503 | 0.114 |
| E31 Satisfaction with the internal environment of community elderly service centres | 4.600±0.503 | 0.109 |
| E32 Satisfaction with age-friendly environment  in the community | 4.600±0.503 | 0.109 |

# 3 The concentration and dispersion of the results corresponding to the second round of Delphi expert consultation

In the second round of consultation, indicators with low importance scores or high coefficients of variation from the first round were eliminated (as shown in Table 3), ensuring the robustness of the final evaluation system,and some indicators were revised and added according to the experts’opinions, and the revised evaluation indicator system was obtained and fed back to the 20 experts.

***Table*** 3 Explanation of Indicator Changes

| **Indicator level** | **Indicator name** | **Modalities of change** |
| --- | --- | --- |
| Third level indicators | A23 Coverage of facilities in community elderly service centres (stations) | Deleted (threshold not reached) |
| Third level indicators | A33 Ratio of the number of carers to the number of older people receiving care in elderly services | Deleted (threshold not reached) |
| Third level indicators | B13 Cost of travelling from home to community care point | Deleted (threshold not reached) |
| Third level indicators | C32 Importance assignment of emotional counselling services | Deleted (threshold not reached) |
| Third level indicators | A33 Ratio of the number of carers to the number of older people receiving care in elderly services | Replaced by A33 Number of service personnel in community elderly service centres (stations) |

The modifications in Table 3 reflect expert feedback to ensure indicator relevance and robustness, particularly in addressing overlapping or redundant metrics.

In the end, the experts' evaluation of the second round of indicator consultation was good, the indicators met the standard requirements, the screening and determination of the indicators was completed, and the evaluation indicator system for the accessibility of basic elderly care services in urban communities was finally established, which contains 5 first-level indicators, 14 second-level indicators and 37 third-level indicators, and the specific contents are shown in Table 4 .

***Table4*** Revised Indicator System: Concentration and Dispersion After Second Round Expert Consultation

| **Indicator Name** | **Importance assignment（**$\bar{\mathbf{X}}$**±S）** | **coefficient of variation**  **（CV）** |
| --- | --- | --- |
| A Availability | 5.000±0.000 | 0.000 |
| A1 Facility Layout | 4.900±0.308 | 0.063 |
| A1 Number of community elderly service  centres (stations) | 4.800±0.410 | 0.085 |
| A12 Building area of community elderly care  service center (station) | 4.900±0.308 | 0.063 |
| A2 Facility configuration | 4.850±0.366 | 0.075 |
| A21 Number of beds in community elderly service  centres (stations) | 4.100±0.308 | 0.075 |
| A22 Number of service facilities in community elderly care service centers (stations) | 4.900±0.308 | 0.063 |
| A3 Resource supply | 4.700±0.571 | 0.121 |
| A31 Types of elderly services provided by community elderly service centres (stations) | 4.150±0.366 | 0.088 |
| A32 The number of elderly care service projects provided by community elderly care service centers (stations) | 4.050±0.224 | 0.055 |
| A33 Number of service personnel in community elderly service centres (stations) | 3.950±0.394 | 0.100 |
| B Accessibility | 4.800±0.410 | 0.085 |
| B1 Space reachable | 4.700±0.470 | 0.100 |
| B11 Distance from residence to community elderly care service center (station) | 4.150±0.366 | 0.088 |
| B12 The convenience level from the residence to the community elderly care service center (station) | 3.900±0.308 | 0.079 |
| B2 Time achievable | 4.800±0.410 | 0.085 |
| B21 Waiting time for nursing staff's on-site service | 4.400±0.681 | 0.155 |
| B22Time consumption from residence to community elderly care service center (station) | 4.400±0.503 | 0.114 |
| C Affordability | 4.950±0.224 | 0.045 |
| C1 Affordability of prices for obtaining life care services | 4.700±0.470 | 0.100 |
| C11 Meal assistance service | 4.500±0.513 | 0.114 |
| C12 Cleaning assistance service | 4.450±0.605 | 0.136 |
| C13 Agency service | 4.300±0.470 | 0.109 |
| C2 Affordable access to medical care services | 4.900±0.308 | 0.063 |
| C21 Rehabilitation nursing services | 4.850±0.366 | 0.075 |
| C22 Health management services | 4.550±0.510 | 0.112 |
| C23 Healthcare Services | 4.350±0.489 | 0.112 |
| C3 Affordability of prices for obtaining mental  comfort services | 4.700±0.470 | 0.100 |
| C31 Emotional Communication Services | 4.250±0.444 | 0.104 |
| C32 Psychological counselling services | 4.300±0.470 | 0.109 |
| C4 Affordability of prices for cultural and  entertainment services | 4.650±0.489 | 0.105 |
| C41 Entertainment | 4.400±0.503 | 0.114 |
| C42 Education for the elderly | 4.400±0.503 | 0.114 |
| D Acceptability | 4.800±0.410 | 0.085 |
| D1 Acceptance of service content | 4.550±0.510 | 0.112 |
| D11 Acceptance of life-care services | 4.750±0.444 | 0.093 |
| D12 Acceptance of medical care services | 4.600±0.503 | 0.109 |
| D13 Acceptance of mental comfort services | 4.650±0.489 | 0.105 |
| D14 Acceptance of cultural and recreational services | 4.350±0.489 | 0.112 |
| D2 Acceptance of service modalities | 4.750±0.444 | 0.093 |
| D21 Acceptance of the home-based care (in-home)  service approach | 4.400±0.503 | 0.114 |
| D22 Acceptance of daycare (day care) services | 4.500±0.513 | 0.114 |
| E Adaptable | 4.950±0.224 | 0.045 |
| E1 Satisfaction with facility construction | 4.800±0.410 | 0.085 |
| E11Satisfaction with the layout of community  elderly service facilities | 4.850±0.366 | 0.075 |
| E12 Satisfaction with the provision of community elderly service facilities | 4.550±0.510 | 0.112 |
| E13 Satisfaction with the ageing-friendly construction of community elderly service centres (stations) | 4.350±0.489 | 0.112 |
| E2 Satisfaction with quality of service | 4.700±0.470 | 0.100 |
| E21 Satisfaction with life care services provided  in the community | 4.750±0.444 | 0.093 |
| E22 Satisfaction with health care services provided  in the community | 4.600±0.503 | 0.109 |
| E23 Satisfaction with mental comfort services provided  in the community | 4.850±0.366 | 0.075 |
| E24 Satisfaction with cultural and recreational services provided by the community | 4.750±0.444 | 0.093 |
| E25 Satisfaction with service personnel | 4.750±0.444 | 0.093 |
| E3 Satisfaction with the service environment | 3.950±0.224 | 0.057 |
| E31 Satisfaction with the internal environment of community elderly service centres | 4.600±0.503 | 0.109 |
| E32 Satisfaction with age-friendly environment  in the community | 4.750±0.444 | 0.093 |

*Note: Importance assignment scores range from 1 to 5, with standard deviations (±S) indicating expert agreement levels.*

# 4 Reliability test

Reliability and validity were confirmed through Cronbach's Alpha (α=0.962) and KMO/Bartlett tests (KMO=0.960, χ²=17568.891, p<0.001), demonstrating strong internal consistency and construct validity.

***Table 5*** Questionnaire reliability tests

| **Cronbach’s α coefficient** | **Item count** | **Sample size** |
| --- | --- | --- |
| 0.962 | 56 | 430 |

***Table 6*** KMO and Bartlett's test

| KMO and Bartlett's test | | |
| --- | --- | --- |
| KMO value | | 0.960 |
| Bartlett Sphericity Check | Approximate chi-square | 17568.891 |
|  | df | 1540 |
|  | p-value | 0.000 |

1. [] Hao, L.; Yan, C.; Yang, X. Development and Evaluation of a Community Elderly Care Service Index System Based on 3C Elements. Chinese Journal of Gerontology 2015, 35(24), 7281–7284. (In Chinese) [↑](#footnote-ref-0)
2. []Yong, L.; Wang, Z.; Zhang, D. Accessibility of Home-Based Elderly Care Community Services: Conceptual Model, Index System, and Comprehensive Evaluation. Population and Economy 2018, (04), 1–11. (In Chinese) [↑](#footnote-ref-1)
3. []Han, X.; Xu, Y. Construction and Empirical Testing of an Accessibility Evaluation Index System for Home-Based Elderly Care Services in Changchun Driven by Data. Chinese Journal of Gerontology 2023, 43(16), 4058–4062. (In Chinese) [↑](#footnote-ref-2)
4. [] Hao, L.; Yan, C.; Yang, X. Development and Evaluation of a Community Elderly Care Service Index System Based on 3C Elements. Chinese Journal of Gerontology 2015, 35(24), 7281–7284. (In Chinese) [↑](#footnote-ref-3)
5. [] Zeng, Q.; Ling, W. Accessibility of Community-Based Integrated Medical and Elderly Care Services: Conceptual Definition, Indicator System, and Comprehensive Evaluation—Based on a Metropolitan Fringe Survey. Theory Monthly 2024, (08), 116–129. (In Chinese) [↑](#footnote-ref-4)
6. [] Xu, X.; Zhao, Y. Spatial Distribution Patterns and Accessibility Evaluation of Elderly Care Facilities in Nanjing: A Two-Step Floating Catchment Area Method Based on Time Costs. Modern Urban Research 2017, (02), 2–11. (In Chinese) [↑](#footnote-ref-5)
7. [] Wu, B.; Wang, X. Optimizing the Accessibility of Elderly Care Facilities from the Perspective of Spatial Justice: A Case Study of Chongqing. Chongqing Social Sciences 2023, (11), 172–188. (In Chinese) [↑](#footnote-ref-6)
8. []Gan, L.; Zhou, L.; Xu, X. Development of a High-Quality Community Home-Based Elderly Care Service Evaluation Index System. China Health Policy Research 2023, 16(07), 40–47. (In Chinese) [↑](#footnote-ref-7)
9. []Li, Y.; Feng, Y. Quality Evaluation of “Embedded Medical and Elderly Care” Services in the Context of Healthy Aging. Journal of Yunnan Minzu University (Philosophy and Social Sciences Edition) 2024, 41(04), 95–105. [↑](#footnote-ref-8)
10. [] Wang, Z.; He, B.; Yong, L. Analytical Framework and Empirical Exploration of the Equalization of Home-Based Elderly Care Community Services in China: Based on Surveys in Beijing, Nanjing, and Xianyang. Scientific Decision Making 2020, (07), 49–69. (In Chinese) [↑](#footnote-ref-9)
11. [] Wang, L.; Feng, Y.; Wang, C. Research on the Service Quality Evaluation of Elderly Care Institutions. Population and Development 2017, 23(06), 96–102. (In Chinese) [↑](#footnote-ref-10)
12. [] Ma, D. Accessibility and Its Influencing Factors of Community Home-Based Elderly Care Services: An Empirical Analysis Based on Survey Data from Five Cities in Two Provinces. Social Security Research 2023, (02), 33–46. (In Chinese) [↑](#footnote-ref-11)
13. [] Zeng, Q.; Ling, W. Accessibility of Community-Based Integrated Medical and Elderly Care Services: Conceptual Definition, Indicator System, and Comprehensive Evaluation—Based on a Metropolitan Fringe Survey. Theory Monthly 2024, (08), 116–129. (In Chinese) [↑](#footnote-ref-12)
14. [] Li, Y.; Feng, Y. Quality Evaluation of “Embedded Medical and Elderly Care” Services in the Context of Healthy Aging. Journal of Yunnan Minzu University (Philosophy and Social Sciences Edition) 2024, 41(04), 95–105. (In Chinese) [↑](#footnote-ref-13)
15. [] Ma, D. Accessibility and Its Influencing Factors of Community Home-Based Elderly Care Services: An Empirical Analysis Based on Survey Data from Five Cities in Two Provinces. Social Security Research 2023, (02), 33–46. [↑](#footnote-ref-14)
